# The Benefit of Conserving and Gaining Resources after Trauma: A Systematic Review

**DOI:** 10.3390/jcm5110104

**Published:** 2016-11-18

**Authors:** Michael Hollifield, Andrea Gory, Jennifer Siedjak, Linda Nguyen, Lucie Holmgreen, Stevan Hobfoll

**Affiliations:** 1 The VA Long Beach Healthcare System, Long Beach, CA 90822, USA; gandrea1011@gmail.com (A.G.); lindaxnguyen19@gmail.com (L.N.); 2 Department of Psychiatry and Behavioral Sciences, University of California at Irvine, Orange, CA 92697, USA; 3 Graduate Department of Psychology, Azusa Pacific University, Azusa, CA 91702, USA; 4 Department of Behavioral Sciences, Rush University Medical Center Chicago, IL 60612, USA; jsiedjak@gmail.com (J.S.); lucie_holmgreen@rush.edu (L.H.); stevan_hobfoll@rush.edu (S.H.)

**Keywords:** trauma, PTSD, resources, interventions

## Abstract

Background: Traumatic events involve loss of resources, which has consistently been found to be associated with developing stress-related illness such as posttraumatic stress disorder (PTSD). Objective: The purpose of this systematic literature review was to determine if there is evidence for the salutatory effect of resource gain on PTSD, and if there are intervention models that utilize and assess gain in PTSD. Data Sources: All relevant online databases were systematically searched using key terms and a method, detailed in Figure 1. Results: Of 22 relevant articles, there were three intervention studies, one longitudinal naturalistic study, eleven non-intervention association studies focusing on PTSD, and eight non-intervention association studies not focusing on PTSD. The intervention and naturalistic studies showed a significant positive effect on PTSD by specifically targeting the gain of resources during an intervention. Other non-intervention research supports the notion that resource loss is pathogenic and resource gain is beneficial after traumatic exposure. Conclusions: Interventions that develop and assess effects of gain of various types of resources on stress-related illness should be encouraged. Interventions that already have proven efficacy for PTSD might include standardized assessment of resource loss and gain to further understand mechanisms of action.

## 1. Introduction

Traumatic events, such as combat, rape, physical assault, natural disasters, and life-threatening illness, often involve the rapid loss of resources [1]. For example, natural disasters often result in loss of shelter, food, and safety, while interpersonal violence may involve loss of the sense of personal security and self-confidence. Lost resources and ongoing stressors strongly predict the risk for developing a stress-related illness following trauma: this has been demonstrated in survivors of war, natural disasters, terrorist attacks, intimate partner violence, and sexual assault [2,3,4,5,6,7,8,9]. For this reason, evaluation of current stressors and loss of resources is recommended during the diagnostic and treatment evaluation for post-traumatic stress disorder (PTSD) [10].

Because of the now clear and consistent evidence that resource loss and ongoing stressors are associated with the development and maintenance of PTSD, it would seem prudent to target and monitor lost resources and objective stressors during treatment. The Conservation of Resources (COR) theory and instruments provide a framework and the tools for this purpose [11,12]. Central to COR theory is the concept that people strive to obtain, retain, and protect the things, or resources, they value. Resources fall into four categories: objects, conditions, personal characteristics, and energies (Hobfoll, 1989) [11]. These resources are highly interrelated, however, and the idea that resource loss contributes to further resource loss is a key aspect of COR theory. Additionally, individuals with less access to resources are more prone to further resource loss under stressful conditions. On the other hand, having more resources can buffer against future resource loss and also facilitate future resource gain (Hobfoll, 1998) [11]. These patterns have been supported through studies showing that material resource loss predicts personal characteristic resource loss in the form of psychological distress [13,14], whereas resource gain is only critical in predicting distress in the context of loss [15].

Recovery and deterioration following a traumatic event illustrate the key COR ideas of “loss spirals” and “gain spirals” [12] so critical in PTSD. In particular, the loss of resources in the aftermath of trauma, when individuals are particularly vulnerable, contributes to the exacerbation of PTSD symptoms in a bidirectional relationship that continues over time [16]. In the aftermath of a natural disaster, for example, individuals’ loss of a home may lead to the subsequent losses of privacy, safety, and social support that come with living in a shelter. Additionally, finances are drained following the natural disaster. Ultimately, optimism decreases. In this example, an initial resource loss (home) contributes to further resource loss (privacy), which continues to affect other resources (optimism).

In spite of research indicating the relationship between resource loss and stress-related illness and the potential benefit of assessing and monitoring resource gain, interventions for stress disorders including PTSD may rely more heavily on appraisal and biological theories with trauma-focused psychotherapies and pharmacotherapy aimed at altering trauma appraisal and biology. The purpose of this review was to determine if there is evidence for the salutatory effect of resource gain during treatment for PTSD. The aim was to specifically determine if there are models of treatment that include the assessment of the possibility that gains consistent with the COR model provide a positive effect on psychopathology (symptoms) and/or functioning (less impairment). This is not a comprehensive review of the benefits of other types of specific gains, not assessed by the COR model, such as the known positive effects of social support of PTSD [17].

## 2. Methods

**Figure 1:**
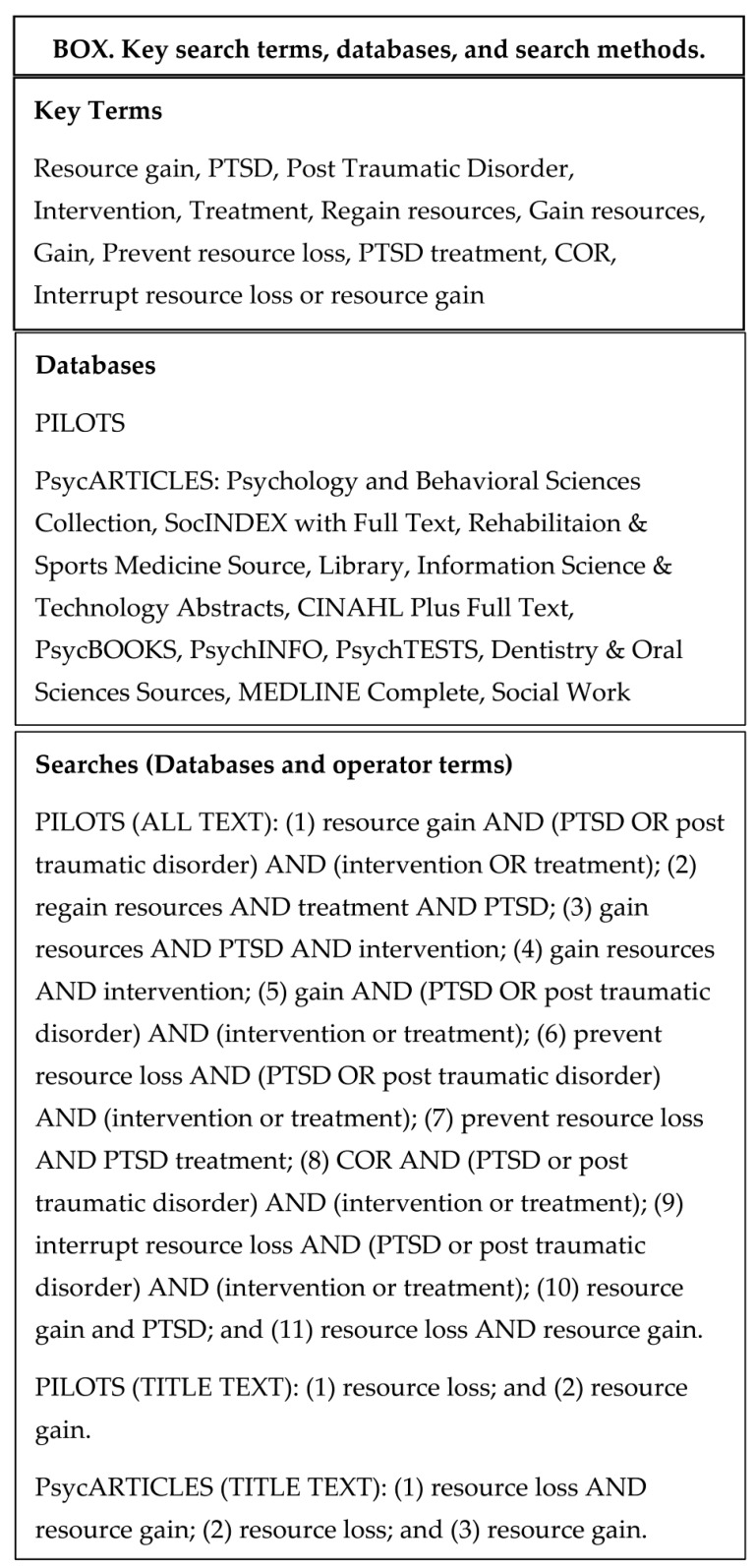
Box about review methodology.

Figure 1(Box) shows relevant key terms chosen, databases searched and the application of key terms to databases. Key terms relevant to topic were first identified. PILOTS and PsycARTICLES were the two databases chosen for the initial search. Key terms were then applied to the databases using two methods: (1) a search with ALL TEXT; and (2) a search within TITLE TEXT. Eleven searches were done in the PILOTS database using combination operators in ALL TEXT. Two searches were completed in the PILOTS database using a search in TITLE TEXT. Three searches were completed in PsycARTICLES using a search in TITLE TEXT. A total of 618 articles were yielded from the search.

One of the authors (A.G.) reviewed all abstracts to identify dissertations and theses, duplicate articles and articles that lacked appropriate content for exclusion. If “gain” was only measured as improvement in symptoms then the article was excluded, since our aim was to identify how resource gain in the COR model might improve symptoms and/or impaired functioning. Abstracts and full PDF articles for all articles were obtained and further review of text was conducted if the abstract was not informative enough to make a decision about inclusion or exclusion. One was in a foreign language and was excluded due to a lack of an English translation. Three authors (A.G., L.N., and J.S.) then reviewed the articles using a form developed specifically for this task to identify thematic categories and article content.

## 3. Results

**Figure 2:**
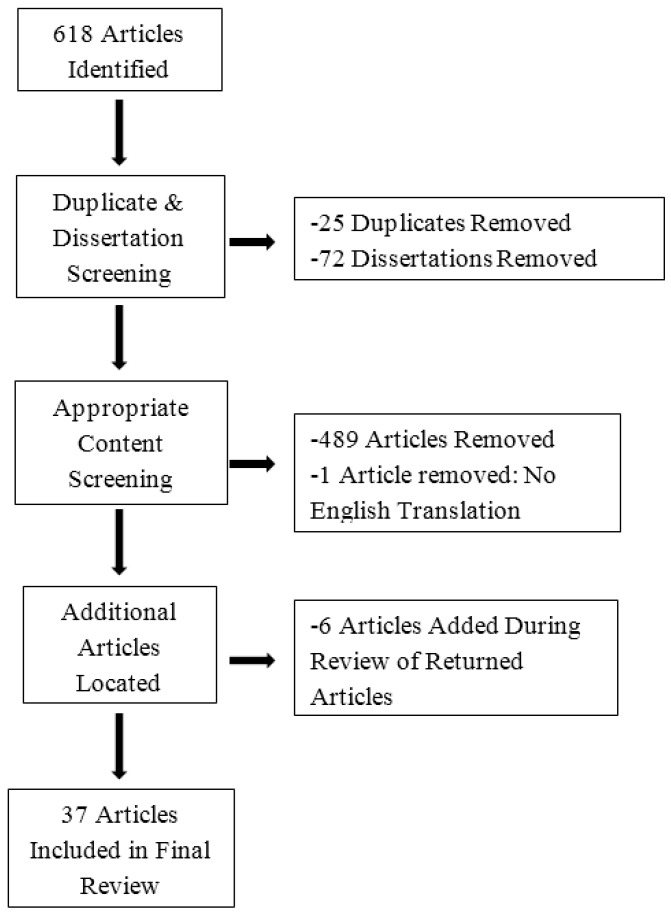
Results of Search.

**Figure 3:**
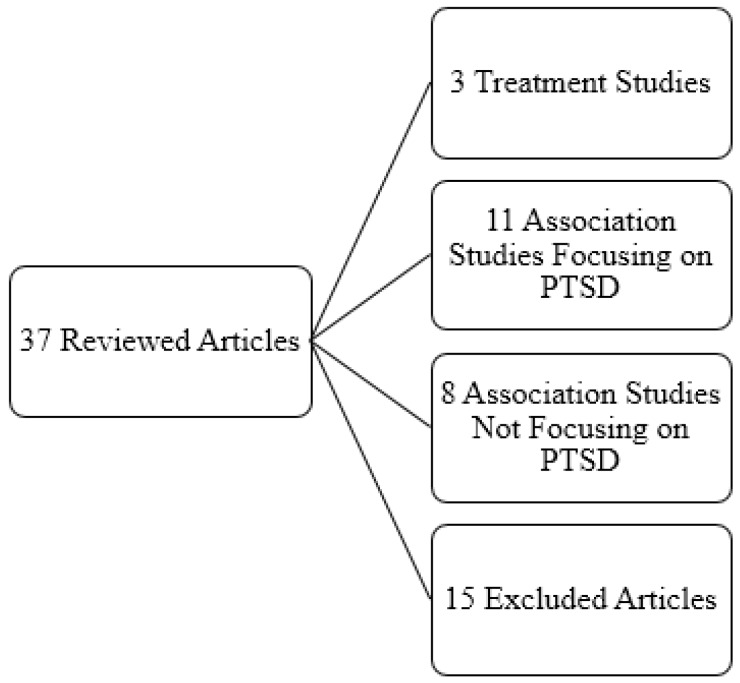
Themes and number of studies included in final review.

Figure 2 and Figure 3 show search results. Of the 618 articles first returned by the search method, 72 were dissertation and theses and 25 were duplicate articles and were thus excluded, one article was excluded for lack of English translation and 489 articles were excluded because of the lack of appropriate content. Six additional articles were found during review of the returned articles and were found to be relevant and were included. Thus, 37 articles comprised the final review. Two authors reviewed all articles and identified four thematic categories: (1) treatment or longitudinal naturalistic studies of which there were three; (2) non-intervention association studies focusing on PTSD, which included 11 articles; (3) non-intervention association studies not focusing on PTSD, eight articles; and (4) articles not applicable to the review, of which there were 15 which were then excluded from further review. Since only three studies provided information about intervention or longitudinal naturalistic models that assessed the effects of resource gain on PTSD, we have also included in the text and tables non-intervention studies that assess the effects of resource gain on symptoms after trauma to broaden the collective information on the subject.

### 3.1. Treatment Studies

Johnson and Zlotnick found that resource gain during and after PTSD treatment was associated with a decrease of PTSD symptoms [18]. Participants included eighteen battered women with PTSD or subthreshold PTSD. Seven also met criteria for major depression, seven for lifetime substance use disorder, and thirteen for other anxiety disorders. The intervention consisted of a cognitive-behavioral therapy named Helping to Overcome PTSD with Empowerment (HOPE). HOPE is a 9–12 session, individual, manualized treatment that addresses the needs of battered women and emphasizes three stages of recovery: (1) establish safety, self-care, and protection; (2) remembrance and mourning; and (3) reconnection. Beginning sessions focus on psychoeducation on interpersonal violence, safety planning and PTSD. Next sessions focus on teaching women information and skills to make informed choices and establishing independence. Later sessions assimilate cognitive behavioral skills to cope with PTSD and PTSD features and address co-occurring problems commonly found among battered women. Participants attended an average of 7.1 sessions of HOPE and reported high satisfaction with the intervention. The therapy was associated with significant improvement in PTSD assessed with the Clinician Administered PTSD Scale (CAPS) (baseline *M* = 60.2, *SD* = 27.2; 6 month post-treatment *M* = 24.0, *SD* = 23.1), depression assessed with the Beck Depression Inventory (BDI) (baseline *M* = 19.4, *SD* = 9.6; 6 month post-treatment *M* = 11.3, *SD* = 11.0), and resource loss assessed with the Conservation of Resources-Evaluation (COR-E) (baseline *M* = 64.7, *SD* = 20.7; 6 month post-treatment *M* = 33.2, *SD* = 33.0). Subjects also reported more effective use of their resources, and improved overall adjustment. The authors did not conduct an interactive analysis of resource loss by symptoms, nor did they describe the type of resource loss that was mitigated by treatment or what resources were gained.

A longitudinal naturalistic study by Walter and Hobfoll found that halting psychosocial and material resource loss through an intervention for sexual behaviors was associated with the abatement of PTSD symptoms [19]. Participants included 102 inner-city women with PTSD from interpersonal trauma. The women were randomly assigned to one of two interventions (HIV/AIDS prevention groups or general health skills and knowledge promotion group) or a usual care control condition (individual sessions that focused on safer sexual and general health behaviors). Resource loss variables (i.e., energy, family interpersonal, non-familial interpersonal, and material resources) were assessed at baseline and 6-month follow-up using the COR-E. At the conclusion of treatment, participants who improved below the diagnostic threshold for PTSD (non-diagnostic; *n* = 59) were compared to participants with PTSD (diagnostic; *n* = 43) (PTSD scores were not provided). At pretest, the groups were not significantly different on resource loss. At the six-month time point, participants with non-diagnostic symptoms reported significantly less resource loss than those with diagnostic PTSD in three of four domains: material, non-diagnostic 4.95, diagnostic, 7.27 (*p* < 0.05); energy, non-diagnostic 7.53, diagnostic 9.84 (*p* < 0.05); family interpersonal, non-diagnostic, 2.44, diagnostic, 4.8 (*p* < 0.001). These findings suggest that trauma recovery is promoted by the continued relationship of family, personal energy resources, and material resources after experiencing a traumatic event.

Thrasher, Power, Morant, Marks and Dalgleish found that a specific type of resource gain, increased social support, on the Significant Others Scale (SOS) significantly predicted symptom improvement on the Clinician Administered Posttraumatic Stress Disorder Scale (CAPS) [17]. Seventy-seven participants aged 16 to 65 years with chronic PTSD completed treatment in a randomized controlled trial (RCT) of exposure therapy (ET) and/or cognitive restructuring (CR), compared with relaxation for adults. The SOS consisted of twelve items about social support in dealing with the effects of trauma. CAPS change score in ET/CR was *M* = 34.8, *SD* = 22.8 compared to *M* = 13.2, *SD* = 23.1 in relaxation. Higher SOS scores were significantly associated with greater improvement in CAPS, *r* = 0.36, *df* = 75, *p* < 0.001. This study was the first to report social support concerning trauma predicting improvement with psychological treatment of PTSD.

### 3.2. Association Studies PTSD Focus

We identified 11 relevant studies that examined the relationship between resource gain and posttraumatic stress symptoms following a traumatic event. Resources were assessed with various instruments. A summary of the findings from these 11 studies is shown in Table 1. The most salient findings about the effects of resource gain on PTSD symptoms were in three studies: Wu, Hung and Chen (2002) [20], Slobodin, Caspi, Klein, Berger and Hobfoll (2011) [21] and Hall, Bonanno, Bolton and Bass (2014) [22]. The other eight studies revealed that experienced resource loss following trauma is associated with negative outcomes such as psychological distress and stress, PTSD symptoms, posttraumatic stress and depressive symptoms.

Wu et al. (2002) found that there were fewer PTSD symptoms in two groups of people, those that experienced “better” resources and those that experienced “no change” of resources after the Chi-Chi Earthquake [20]. These two groups were also equipped with better coping resources than the groups that experienced “worse” resources after the earthquake. Slobodin and colleagues found that Bedouin servicemen who experienced resource gain were more able than those who did not gain resources to effectively use defensive and avoidant strategies [21]. Authors opined that by gaining this ability, servicemen may be able to engage in less aversive thoughts and memories of their experienced trauma, further empowering them to gain more object resources following the traumatic experience. Loss of personal resources mediated the impact of trauma exposure on symptoms. Hall et al. (2014) found that male and female torture survivors experienced decreased depression and PTSD symptoms in relation to gaining social contact and social integration [22]. The authors argued that there is an “intuitive” inverse relationship between social resources and psychological distress. As distressing symptoms decrease, social resources improve. Equally, as gains in social resources occur, symptoms of distress would lessen.

### 3.3. Association Studies No PTSD Focus

Six studies found an association between experiencing resource gain and improvement of non-PTSD clinical symptoms after experiencing trauma: a summary of the results of these studies is shown in Table 2. Examples of experienced resource gain include social support, active coping, optimistic feelings of self and others, feeling life has meaningful purpose, psychosocial resources, materials, mastery, religious comfort and sense of cohesion. Positive impacts on clinical symptoms included decreased anger, depression, anxiety, and psychological dysfunction. Other positive associations from resource gain include positive adjustment after trauma and buffering of negative effects of resource loss on emotional health.

One study showed that resource gain had no effect on clinical symptoms and another study examined resource loss but not gain. Zwiebach and colleagues reported that resource gain had no effect in their study of patterns of loss, gain and subsequent mental health in 402 Hurricane Katrina survivors. In a hierarchical regression model, gains made in social support, future orientation, goal orientation, physical health, and health insurance were not associated with changes in psychological distress [23].

## 4. Discussion

The aim of this review was to determine whether there are models of treatment that include the assessment of the relationship between gains consistent with the COR model and have a positive effect on psychopathology and/or functioning. Because the literature on this specific topic was found to be sparse, we broadened the review to include non-intervention research that would add knowledge about the possible effect of resource gain on PTSD symptoms and/or functioning. There is only one published study found that showed a significant positive effect on PTSD by specifically targeting lost resources during intervention. A second study focusing on reducing psychosocial and material resource loss through an intervention for sexual behaviors demonstrated a reduction in PTSD associated with the reduction of loss, and a third demonstrated that increasing social support was associated with lessening PTSD symptoms. These studies, however, evaluated the interaction between intervention and reduction of lost resources rather than the presence of an actual gain of resources that helped ameliorate symptoms and functioning. Whether reduction of lost resources is similar or the same to gained resources in content and effects on treatment remains an open question. These three studies, nonetheless, build on and operationalize the literature synthesized in this review showing that loss of resources following traumatic stress is a significant predictor of PTSD and other clinical conditions, and reducing lost resources is salutary for PTSD and other clinical conditions. Other non-intervention research supports the idea that resource loss is pathogenic and resource gain, or at least reduction of loss, is salutary after traumatic exposure. A methodological point is that there were many assessment instruments used, which are referenced in the Appendix A. While recommending a standardized approach is not central to the current work, the field may benefit from such standardization to enhance comparative research.

Findings from this review indicates that evidence-based treatment for PTSD is aimed primarily at pathology reduction in the individual and does not commonly assess the role of objective stressors, resource loss, or resource gain as mechanisms of action. Interventions that have evidence of efficacy for PTSD include a group of therapies under the rubric of cognitive behavior therapy (CBT) such as prolonged exposure, cognitive processing therapy and stress inoculation, eye movement desensitization and reprocessing, and various pharmacological therapies as monotherapy or combined with CBT [10,38,39]. There is very little research about the mechanisms of action of these therapies [40]. This literature implies that the effect of the gain of resources has a significant part on altering symptoms and/or the biology within the individual For example, exposure therapy appears to have large effects on anxiety and fear, but not much is known about its effects on avoidance, social isolation, interpersonal problems, or anger [41]. While altering anxiety and fear responses is critical to trauma recovery, the broader mechanisms related to positive resource gain of many types is poorly understood and warrants further study.

There are undoubtedly studies about interventions that promote resource gain that were not found in this literature review due to inherent limitation of electronic searching. For example, Trauma Management Therapy (TFT) for PTSD was developed for combat Veterans and showed that a combination of education, exposure, and social and emotional skills training decreased symptoms and increased participation in social and employment activities [42]. In another study primarily about the benefit of acupuncture for PTSD, the comparator intervention was a 12-week integrated CBT delivered in group [43]. Sessions 1 to 3 utilize psychoeducation, behavioral activation, and activity planning, collectively termed “Trajectory and Resource Loss Stabilization”. Participants identify valued resources that have either been lost or are at-risk, and then make plans to engage in activities that will help establish a resource gain cycle. During Sessions 4 to 12 where participants are taught classic cognitive restructuring [44], imagery rehearsal [45], and exposure and desensitization techniques [46,47], participants continue to establish a resource gain cycle. Simple effects analyses showed that symptoms on the Post-Traumatic Symptom Scale—Self-Report (PSS-SR) declined significantly from baseline to end-treatment, with 36% having PSS-SR scores below the entry criterion level of ≥16 at end-treatment.

Other interventions that have at least some evidence of efficacy, such as patient education, relaxation, imagery rehearsal therapy, brief psychodynamic therapy, hypnosis, and acupuncture, most likely help the patient with PTSD gain resources of some type(s), yet assessment of that gain is poorly understood while symptom reduction as a primary and worthy goal is measured. The VA/DOD guidelines describe many types of interventions of psychosocial rehabilitation and spiritual support under “Adjunctive Services”, many of which undoubtedly help PTSD sufferers re-gain valuable lost resources. Clinical guidelines soundly describe the need to assess symptoms and functioning at many points along the triage-diagnosis-intervention trajectory, yet recommending evaluation of resources that are at risk or lost and those resources that are gained with treatment is absent [10]. A group of scholars convened to develop essential elements of intervention for the immediate and mid-term period after mass trauma, focusing on identifying principles that have empirical support for promoting resistant and resilient outcomes. The five principles identified were to promote: (1) a sense of safety; (2) calming; (3) a sense of self- and collective-efficacy; (4) connectedness; and (5) hope [48]. These principles if applied favor targeting the gain of internal and external resources over primary symptom reduction. While there are no empirical data on the effectiveness of applying these principles in a clinical setting, they show promise as a general and standard method to mitigate loss and promote gain. It would also help the field if assessment and treatment guidelines would recommend assessing resources lost and gains made during treatment.

Traumatic events rapidly change the trajectory of one’s lived experience, and not only significantly alter cognitive and biological functioning, but also one’s identity and sense of belonging in a world that has to the survivor become different than before the events. Multiple types of available resources have been lost to the survivor with PTSD and other distressing symptoms. A synthetic view using COR theory and data about the relevance of resource loss associated with illness and gain associated with improvement would suggest that symptoms and functioning are one of many resource types that should be evaluated pre- and post-treatment. These findings should encourage the field of traumatic stress to develop interventions that assess the effects of the gain of various types of lost or at-risk resources on PTSD and other stress-related illness, and to include and standardize assessment of resource loss and gain in research using interventions that have proven efficacy for PTSD.

## Figures and Tables

**Table 1:** Association Studies with a PTSD Focus, Summary Table.

Authors and Year	Research Question	Sample size, Population, and Trauma Experience	Resources Measured	Other Measured Clinical Symptoms	Experienced Resource Gain	Positive Associations with Resource Gain	Associations with Resource Loss
Wu, Hung, Chen, 2002 [20]	Is there a positive relationship between changes of health conditions/social relationships and the state of possessed resources?	556 adults Taiwan Chi-Chi Earthquake	Objective and Subjective Threat ^1^ Subjective Evaluation of changes in life domains and coping resources ^2^ Coping Resources ^4^	Severity of Posttraumatic Symptoms ^3^	Socio-economic Domain Social-interpersonal Domain Personal health Domain Overall Condition	Groups that experience “better” or “no change” in resources displayed fewer PTSD symptoms.	“Worse” group reported more posttraumatic symptoms.
Slobodin, Caspi, Klein, Berger and Hobfoll, 2011 [21]	Does loss of resources mediate the relationship between trauma and posttraumatic responses?	317 Bedouin servicemen Israeli Defense Forces	COR-E ^5^ Traumatic Events ^6^	Depression ^7^ Anxiety ^7^	Trauma-related gains in object resources Trauma-related gains in material resources	Gains reflected the ability to use defensive and avoidant strategies effectively.	Loss of personal resources negatively mediates the impact of trauma on development of psychological symptoms.
Hall, Bonanno, Bolton and Bass, 2014 [22]	Does baseline psychological distress symptoms and changes in these symptoms were associated with changes in social resources?	96 male and female adults Torture survivors	Social Support ^30^ Social Integration ^66^ Frequency of Social Contact ^67^ Demographic Variables ^8^	Depression ^7^ Anxiety ^7^ Posttraumatic Stress Symptoms Traumatic Grief	Social Support Social Integration Frequency of Social Contact	Decreased depression, anxiety and PTSD symptoms was significantly associated to gaining social integration. Decreased depression and PTSD was associated to gaining social contact.	Depression symptoms, PTSD symptoms and traumatic grief were associated with losses to social integration.
Hobfoll, Canetti-Nisim and Johnson, 2006 [24]	What is the impact of terrorism on PTSD symptoms and depressive symptoms?	905 Jewish and Palestinian citizens of Israel Al Aqsa Intifada acts of terrorism	Demographic Variables ^8^ Terrorism Exposure ^9^ Economic Resources ^10^ Psychological Resources ^11^ Support Satisfaction ^12^ Protective Attitudes ^13^	Depressive Symptoms ^14^ PTSD ^15^	Psychological Resource Gain		Psychosocial resource loss is strongly related to PTSD symptoms and depressive symptoms.
Heath, Hall, Russ, Canetti and Hobfoll, 2012 [25]	What are the transitional relationships among resource loss and psychological distress?	752 Palestinian adults Political Violence	Intrapersonal and Interpersonal Resources ^5^	PTSD ^17^ Depressive Symptoms ^18^			Psychological resource loss and psychological distress predicted each other over time.
Blaze and Shwalb, 2009 [26]	What are the long term psychological impacts of Hurricane Katrina?	636 high school aged students Hurricane Katrina	Resource Loss ^19^ Self-Esteem ^20^ Optimism ^21^	General Psychological Distress (GPD) ^22^ Posttraumatic Stress (PTS) ^23^			Self-esteem scores predicted posttraumatic stress. Lower self-esteem, lower optimism, and greater relocation strongly predicted GPD.
Littleton, Grills-Taquechel and Axsom, 2009 [27]	What are the risk factors for posttraumatic symptomatology?	293 female university students Mass shooting at Virginia Tech	Social Support ^24^ Resource Loss ^5^	PTSD ^25^ Depression ^26^ Anxiety ^27^			Resource loss in the time after shootings predicted experienced trauma.
Hobfoll, Tracey and Galea, 2006 [28]	Does resource loss predict PTSD and depression?	2752 random individuals in New York City 11 September 2001 World Trade Center	Sociodemographic characteristics ^8^ 9/11 experiences ^28^ Lifetime Trauma Event Exposures ^29^ Social Support ^30^ Resource Loss and Resource Gain ^5^	Panic Attack Symptoms ^31^ PTSD ^32^ Depression ^33^			Critical role of resource loss in predicting PTSD and depression following terrorism.
Dekel and Hobfoll, 2007 [29]	Examine emotional adjustment of Holocaust survivors when facing new stressors (Intifada).	102 Holocaust survivors of Israel Terror and threat of missile attack	Loss of Personal Resources ^11^ Loss of Interpersonal Resources ^11^	PTSD ^16^ Psychiatric Symptoms ^34^			During Holocaust those who lost a spouse or child had higher PTSD symptoms During Intifada higher loss of interpersonal psychological and person resources was strongly associated with higher PTSD.
Cordova, Walser, Neff and Ruzek, 2005 [30]	To identify factors that influence emotional adjustment after injury to prevent future psychological impairment.	47 emergency room admitted patients Traumatic Experience	COR-E ^5^ Social Constraints ^35^ Stressfulness of Event ^36^ Acceptance and Action ^37^ Demographics ^8^	PTSD ^38^ Depression ^26^			Greater social constraints were associated with greater PTSD symptoms.
Littleton, Kumpula and Orcutt, 2011 [31]	Do psychosocial resources predict PTSD symptoms?	691 college women Mass shooting at Northern Illinois University	Life Trauma History ^39^ Exposure to Shooting ^41^ Resource Loss ^5^	PTSD ^42^ Psychological Distress ^40^			Resource loss experienced in the aftermath of the campus shooting predicted PTSD symptoms.

1–67: See Appendix A.

**Table 2:** Association Studies without PTSD Focus, Summary Table.

Authors and Year	Study	Sample Population and Trauma Experienced	Resources Measured	Other Measured Clinical Symptoms	Experienced Resource Gain	Associations of Resource Gain
Littleton, Axsom and Grills-Taquechel, 2009 [32]	Longitudinally examine interpersonal and interpersonal resource loss and gain in relation to college students’ psychological distress.	193 College Women; ages 18–27 Virginia Tech mass shooting	Resource Loss and Resource Gain ^5^ Social Support ^24^ Coping Strategies ^43^ Exposure to Shooting Incident ^41^	Depression ^26^ Anxiety ^27^	Social Support Active Coping	Decrease in depression and anxiety symptoms.
Wells, Hobfoll and Lavin, 1999 [33]	Pattern of resource loss and resource gain in pregnant women during pregnancy and following pregnancy.	71 women Pregnancy	COR-E ^5^	Depressive Mood ^26^ Anger ^44^	Gains during and after pregnancy include increase in self pride, optimism, and better relationship with others	Decrease in anger and depressed mood.
Hobfoll, Johnson, Ennis and Jackson, 2003 [34]	Study how economic stress (material loss) alters women’s personal and social resources and how changes in the resources impact anger and depressive mood.	714 women Inner City	Material Loss ^5^ Mastery ^45^ Social Support ^12^	Depressive Mood ^46^ Anger ^47^	Mastery Gain Material Gain Social Support	Mastery gain has less depressive mood. Decrease of material loss experienced lower depressed mood and anger.
Holahan, Moos, Holahan and Cronkite, 1999 [35]	Better understand the role of psychosocial resources in the stress and coping process.	326 Individuals Community Sample	Sociodemographics ^8^ Family Support ^48^ Personality Characteristics ^49^ Life-Change Events ^50^	Depressive Symptoms ^51^	Psychosocial Resources	Decrease of depressive symptoms Decrease of excess negative life events.
Cook, Aten, Moore, Hook and Davis, 2013 [36]	Examine associations among resource loss, religiousness, posttraumatic growth, and physical and mental health.	189 college students Hurricane Katrina	Resource Loss ^11^ General Religiousness ^52, 53^ Health and Adjustment ^54^ Posttraumatic Growth ^55^		Religious Comfort	Positive adjustment Buffered negative effects of resource loss on emotional health.
Ying, Akutsu, Zhang and Huang, 1997 [37]	Test if sense of coherence serves as a mediator between stressors, resources, and psychological functioning.	2234 Vietnamese, Cambodian, Laotian, Hmong, and Chinese-Vietnamese refugees	Resistance Deficit Variables ^56^ Sense of Coherence ^57^ Psychological Dysfunction ^58^	Depression ^58^ Anxiety ^58^	Sense of Cohesion	Lower rates of depression, anxiety and psychosocial dysfunction.
Zwieback and et al., 2010 [23]	Analyzed patterns of loss, and gain and subsequent mental health.	402 survivors of Hurricane Katrina	Social Support ^60^ Participants Outlook Goal Orientation ^61, 62^ Health and Hurricane Exposure ^63^	Psychological Distress ^59^	Social Support Future Orientation Physical Health Insurance	Resource gain showed no effect.
Smith and Freedy, 2000 [9]	Examine the role of psychosocial resource loss after the Midwest Flood. The studied resources fully mediated flood effects.	131 adults Midwest Flood	Flood Exposure ^64^ Psychosocial Resources ^11^ Psychological Distress ^22^ Physical Symptoms ^65^		Resource gain was explained.	

43–65: See Appendix A.

## Appendix A

**Table A1:** Various assessment instruments used to measure constructs.

	Instrument	Construct Assessed	* Validated or Self-Made
1.	Objective and Subjective Index of Threat Questionnaire	Objective and Subject Threat	Self-Made
2.	Changes in Life Domain Questionnaire	Subjective Evaluation of Changes In Life Domains and Coping Resources	Self-Made
3.	The Posttraumatic Stress Reaction Index	Severity of Posttraumatic Symptoms	Validated
4.	Coping Resources Questionnaire	Coping Resources	Self-Made
5.	Conservation of Resources—Evaluation (COR-E)	Resources	Validated
6.	Diagnostic and Statistical Manual of Mental Disorders (DSM) IV Criterion A for PTSD	Traumatic Events	Validated
7.	Hopkins Symptom Checklist-25 (HSCL-25)	Anxiety and Depression	Validated
8.	Demographic Questionnaire	Demographic Variables	Self-Made
9.	Exposure to Terrorist or War-Related Event Questionnaire	Terrorist Attacks or War Related Events Since Beginning of Al Aqsa Intifada	Self-Made
10.	Economic Resources Questionnaire	Loss of Economic Resources	Self-Made
11.	COR Theory Questionnaire	Resources	Self-Made
12.	Social Support Questionnaire	Support Satisfaction	Validated
13.	Ethnocentrism, Political Violence, and Authoritarianism Questionnaire	Protective Attitudes	Self-Made
14.	Public Health Questionnaire	Depressive Symptoms	Validated
15.	Abbreviated Version of PTSD Symptom Scale	PTSD	Self-Made
16.	PTSD Inventory	PTSD	Validated
17.	PTSD Symptom Scale-Interview Format (PSS-I)	PTSD	Validated
18.	Patient Health Questionnaire (PHQ-9)	Depressive Symptoms	Validated
19.	Resource Loss Scale for Children (RLSC)	Resource Loss	Validated
20.	Self-Esteem Inventory	Self-Esteem	Validated
21.	Life Orientation Test	Optimism	Validated
22.	General Health Questionnaire-12	General Psychological Distress	Validated
23.	Impact of Events Scale—Revised (IES-R)	Posttraumatic Stress	Validated
24.	Multidimensional Scale of Perceived Social Support (MSPSS)	Social Support	Validated
25.	PTSD Symptoms Scale—Self Report (PSS-SR)	PTSD	Validated
26.	Center for Epidemiologic Studies Depression Scale (CES-D)	Depression	Validated
27.	Four Dimension Anxiety Scale (FDAS)	Anxiety	Validated
28.	September 11th Event Experiences Questionnaire	9/11 Experiences	Self-Made
29.	Lifetime Trauma Event Exposures Questionnaire	Lifetime Trauma Event Exposures	Self-Made
30.	Social Support Questionnaire	Social Support	Self-Made
31.	DSM IV Criteria for Panic Attacks	Panic Attack Symptoms	Validated
32.	National Women’s Study (NWS) PTSD Module	PTSD	Validated
33.	Major Depressive Scale from Structured Clinical Interview for DSM III—Revised	Depression	Validated
34.	Brief Symptom Inventory (BSI)	Psychiatric Symptoms	Validated
35.	Social Constraints Scale (SCS)	Social Constraints	Validated
36.	PTSD Stressor Criteria DSM-IV	Stressfulness of Events	Validated
37.	Acceptance and Action Questionnaire (AAQ)	Acceptance and Action	Validated
38.	Post-Traumatic Stress Disorder Checklist—Civilian (PCL-C)	PTSD	Validated
39.	Life Trauma History	Traumatic Life Events Questionnaire (TLEQ)	Validated
40.	Depression Anxiety Stress Scale 21 (DASS-21)	Psychological Distress	Validated
41.	Exposure to Shooting Incident Questionnaire	Exposure To Shooting	Self-Made
42.	Distressing Events Questionnaires (DEQ)	PTSD	Validated
43.	Coping Strategies Inventory (CSI)	Coping Strategies	Validated
44.	State Trait Anger Scale (STAS)	Anger	Validated
45.	Mastery Scale	Mastery	Validated
46.	Profile of Mood States (POMS)	Depressive Mood	Validated
47.	State-Trait Expression Inventory (STAXI)	Anger	Validated
48.	Family Environment Services (FES)	Family Support	Validated
49.	Personality Characteristics Questionnaire	Personality Characteristics	Self-Made
50.	Serious Negative Life Events Questionnaire	Life-Change Events	Validated
51.	Research Diagnostic Criteria—Depressive Symptoms Index	Depressive Symptoms	Validated
52.	Duke Religious Index (DRI)	General Religiousness	Validated
53.	Religious Comfort and Strain Scale (RCSS)	General Religiousness	Validated
54.	RAND Health Survey	Health and Adjustment	Validated
55.	Posttraumatic Growth Inventory	Posttraumatic Growth	Validated
56.	Resistance Deficit Variables	Generalized Resistance Resources	Self-Made
57.	Sense of Coherence Scale	Sense of Coherence	Validated
58.	Florida Health and Family Life Instrument	Psychological Dysfunction	Validated
59.	K6 Scale	Psychological Distress	Validated
60.	Social Provisions Scale	Social Support	Validated
61.	Outlook and Sense of Identity Questionnaire	Goal Orientation	Self-Made
62.	Reactive Responding—Short Form	Goal Orientation	Validated
63.	Health and Hurricane Questionnaire	Health and Hurricane Exposure	Self-Made
64.	Flood Exposure Questionnaire	Flood Exposure	Validated
65.	Physical Symptoms Index	Physical Symptoms	Validated
66.	Social Integration Questionnaire	Social Integration	Self-Made
67.	Frequency of Social Interaction Questionnaire	Frequency of Social Interactions	Self-Made

* “Validated” refers to there being support in the literature for the instrument having been shown valid with metric testing. “Self-made” refers to the instrument being made de-novo for the study cited.
